# Genome-guided analysis of physiological and morphological traits of the fermentative acetate oxidizer *Thermacetogenium phaeum*

**DOI:** 10.1186/1471-2164-13-723

**Published:** 2012-12-23

**Authors:** Dirk Oehler, Anja Poehlein, Andreas Leimbach, Nicolai Müller, Rolf Daniel, Gerhard Gottschalk, Bernhard Schink

**Affiliations:** 1 Department of Biology, Microbial Ecology, University of Konstanz, Konstanz, D-78457, Germany; 2 Genomic and Applied Microbiology and Göttingen Genomics Laboratory, Georg-August University Göttingen, Göttingen, D-37077, Germany; 3 Department of Microbiology and Institute for Genomic Biology, University of Illinois, 601 S. Goodwin, Urbana, IL, 61801, USA

## Abstract

**Background:**

*Thermacetogenium phaeum* is a thermophilic strictly anaerobic bacterium oxidizing acetate to CO_2_ in syntrophic association with a methanogenic partner. It can also grow in pure culture, e.g., by fermentation of methanol to acetate. The key enzymes of homoacetate fermentation (Wood-Ljungdahl pathway) are used both in acetate oxidation and acetate formation. The obvious reversibility of this pathway in this organism is of specific interest since syntrophic acetate oxidation operates close to the energetic limitations of microbial life.

**Results:**

The genome of *Th. phaeum* is organized on a single circular chromosome and has a total size of 2,939,057 bp. It comprises 3.215 open reading frames of which 75% could be assigned to a gene function. The G+C content is 53.88 mol%. Many CRISPR sequences were found, indicating heavy phage attack in the past. A complete gene set for a phage was found in the genome, and indications of phage action could also be observed in culture. The genome contained all genes required for CO_2_ reduction through the Wood-Ljungdahl pathway, including two formyl tetrahydrofolate ligases, three carbon monoxide dehydrogenases, one formate hydrogenlyase complex, three further formate dehydrogenases, and three further hydrogenases. The bacterium contains a menaquinone MQ-7. No indications of cytochromes or Rnf complexes could be found in the genome.

**Conclusions:**

The information obtained from the genome sequence indicates that *Th. phaeum* differs basically from the three homoacetogenic bacteria sequenced so far, i.e., the sodium ion-dependent *Acetobacterium woodii,* the ethanol-producing *Clostridium ljungdahlii*, and the cytochrome-containing *Moorella thermoacetica*. The specific enzyme outfit of *Th. phaeum* obviously allows ATP formation both in acetate formation and acetate oxidation.

## Background

Methanogenesis is the dominant process of organic matter degradation in anoxic habitats in the absence of alternative inorganic electron acceptors such as nitrate, manganese, iron, or sulfate. In the overall electron flow, acetate is the most important precursor of methane [1-6]. Acetate is converted to methane through two different pathways: either by aceticlastic methanogenesis as carried out by *Methanosarcina* or *Methanosaeta* spp. [6,7], or by syntrophic acetate oxidizers which depend on close cooperation with hydrogenotrophic methanogens [8,9].

In natural environments, syntrophic acetate oxidation was observed so far only in rice field soil or in subtropical lake sediments [10,11]. Up to this point, only six syntrophic acetate oxidizers were isolated in defined co-cultures, and all of them were obtained from anaerobic digesters [9,12-14]. Syntrophic acetate oxidation may outcompete aceticlastic methanogenesis especially at enhanced temperature, enhanced proton activity, or high acetate concentrations, which all help to improve the energetically difficult situation of syntrophic acetate oxidation [15]. Moreover, ammonia at enhanced concentrations inhibits aceticlastic methanogens and thus selects for syntrophic acetate oxidizers, e. g. in digesters treating nitrogen-rich wastes such as manure [16-20].

*Thermacetogenium phaeum* was isolated from sludge of an anaerobic digester run at 58°C. It was described as a rod-shaped, spore-forming and motile bacterium able to grow with various substrates such as alcohols and methylated nitrogen compounds, and to reduce sulfate in the presence of acetate. In cooperation with *Methanothermobacter thermautotrophicus, Th. phaeum* degrades acetate as sole carbon source [13,21]. Enzymes of the Wood-Ljungdahl (CO dehydrogenase) pathway are active in cells grown either syntrophically with acetate or in pure culture with methanol [22].

In comparison with the mesophilic syntrophic acetate oxidizer *Clostridium ultunense* strain BS (doubling time 480–600 h) [23], *Th. phaeum* grows rapidly (doubling time 69–76 h) in syntrophic co-culture with acetate; 40 mM acetate is degraded in 24 days. Enhanced growth at elevated temperature can be explained by the concomitant gain in free energy [15]. In pure culture, *C. ultunense* cannot grow with methylamine or alcohols, but grows with glucose, betaine, or ethylene glycol. *Th. phaeum* cannot degrade these compounds [12,13].

Until now, all reported syntrophic acetate oxidizers were described as facultative homoacetogens. There are two metabolic types of homoacetogenic bacteria, the proton-dependent (e. g., *Moorella thermoacetica,* formerly *Clostridium thermoaceticum*) and the sodium-dependent acetogens *(*e. g., *Acetobacterium woodii);* both types use the Wood-Ljungdahl pathway for acetate formation [24]. *M. thermoacetica* contains a menaquinone-7 and two *b*-type cytochromes [25] which are assumed to be involved in energy conservation. Enzyme assays with membrane vesicles showed that a hydrogenase, a carbon monoxide dehydrogenase, a methylene-tetrahydrofolate (H_4_F) reductase, and a NADH dehydrogenase are membrane-associated and likely to be involved in energy conservation [26]. In addition, genome sequencing found indications of a membrane-bound formate hydrogenlyase system [27].

The genome of *Acetobacterium woodii* has recently been sequenced [28]. This bacterium can be considered as the model organism of sodium-dependent acetogens. In contrast to *M. thermoacetica, A. woodii* does not contain cytochromes or quinones. Rather energy appears to be conserved through a membrane-bound Rnf complex [29]. *Clostridium ljungdahlii*, a homoacetogenic bacterium producing ethanol as a side product, represents a third option of energy conservation: it does not contain cytochromes, is independent of sodium ions, but contains a proton translocating Rnf complex [30].

In the recent past, several genomes of strict anaerobes have been sequenced which are involved in syntrophic oxidation of butyrate (*Syntrophomonas wolfei*), benzoate (*Syntrophus aciditrophicus*), or propionate (*Syntrophobacter fumaroxidans*, *Pelotomaculum thermopropionicum*) [31-34]. In all these types of syntrophic metabolism, certain oxidation steps at comparably positive redox potentials are involved, e. g., oxidation of butyryl-CoA to crotonyl-CoA, or of succinate to fumarate. The electrons released in these reactions require a reversed electron transport to be raised to the redox potential of the proton/hydrogen pair (E’ about −300 mV at pH 7.0 and [H_2_ at 10^-4^ atm.).

In this study, we report the analysis of the complete genome of the syntrophically acetate-oxidizing bacterium *Th. phaeum*, which is studied as an example of a metabolism operating close to thermodynamic limits. The results of the genome analysis could be verified in physiological experiments.

## Methods

### Microorganism and growth conditions

*Thermacetogenium phaeum* strain PB (DSM 12270) and *Methanothermobacter thermautotrophicus strain TM* were obtained from the DSMZ, Braunschweig, Germany. The composition of the basal medium used for all experiments has been described previously [13].

### Determination of growth

Cultures were incubated at 60°C in 100 ml serum bottles containing 100 ml medium, growth was determined by measuring OD_600_ with a spectrophotometer (Uvikon 860, Kontron Instruments). All growth experiments were performed at least in duplicate. Substrates were added from filter-sterilized or autoclaved anoxic stock solutions to the desired final concentrations. Gases were supplied in the headspace, either as a H_2_/CO_2_ mixture (80/20, v/v, 130 kPa) or a CO/CO_2_/N_2_ mixture (20/20/60; v/v/v, 130 kPa). Concentrations of substrates used in growth experiments were 20 mM methanol, 5 mM formate, 10 mM acetate, 10 mM sulfate, 10 mM thiosulfate, 20 mM acetoin, 20 mM 2,3-butanediol, 20 mM propanol, 20 mM ethanol, 20 mM trimethylamine, and 20 mM syringate.

### Sequencing strategy

Genomic DNA of *T. phaeum* was isolated using the MasterPure™ complete DNA purification kit (Epicentre, Madison, Wi., USA). The extracted DNA was used to generate 454 shotgun and paired-end libraries according to the manufacturer’s protocols (Roche 454, Branford, USA). In each case, one quarter lane of a Titanium picotiter plate was used for sequencing of the libraries, resulting in 506605 total reads with 113390 paired reads. The reads were *de novo* assembled using the Roche Newbler assembly software 2.3 (Roche 454). Closure of remaining gaps and sequence polishing were done by PCR-based techniques and Sanger sequencing of PCR products using BigDye 3.0 chemistry and avn ABI3730XL capillary sequencer (Applied Biosystems, Life Technologies GmbH, Darmstadt, Germany). The Gap4 (v.4.11) software of the Staden package (Staden, 1998) was used for sequence editing.

### Gene prediction and annotation

Automatic gene prediction was performed with the YACOP and GLIMMER [35] software packages. All predicted genes were manually corrected based on GC frame plot analysis, the presence of ribosome-binding sites, and comparison to known protein-encoding sequences employing the Sanger Artemis tool v13 [36]. Functional annotation was initially carried out with the ERGO software suite [37] and the IMG/ER (Integrated Microbial Genomes/Expert Review) system [38]. Subsequently, the annotation was manually curated by comparison to the Swiss-Prot, TrEMBL, and InterPro database [39,40].

### Sequence analysis and comparative genomics

Gene products were classified into functional categories performing a BLAST search against the COG database [41]. A bidirectional BLAST algorithm was used for comparative analyses of different organisms as described previously [42], combined with a global sequence alignment based on the Needleman-Wunsch algorithm [43]. Genes were assumed to be orthologs at a global alignment similarity of higher than 30% and a BLAST e-value lower than 10e-21. Visualization of the chromosome and other DNA sequences was done with DNAPlotter [44]. The pathway tool software from the BioCyc Database collection [45] was employed to analyze metabolic pathways. The reconstruction and validation of metabolic pathways was curated manually.

The multiple sequence alignment was perfomed with the ClustalW [46] and default settings were used. TAT motif prediction was performed with PRED-TAT [47].

### Assay of motility

Motility was tested with two different methods. First, the motility of free-swimming cells was assayed under oxic conditions immediately after taking samples. Second, in order to force *Th. phaeum* to express potential flagella, swarm agar tubes were prepared [48]. Gelrite (0.4%, 0.6% and 1.0% w/v) was used instead of agar, and 20 mM methanol was added as energy and carbon source.

### Detection of gas vesicles

Gas vesicles were searched for by phase-contrast microscopy. For further investigation of light-diffracting structures inside the cells, a 1-ml culture was exposed to high pressure (150 bar) to destroy possible gas vesicles.

### Detection of phages

A 50 ml pure culture grown with 20 mM methanol as substrate was centrifuged at 5,000 x *g* for 20 min. The supernatant was transferred to a 50 ml Falcon tube containing 1/6 volume of a 20% (w/v) PEG 6000 plus 2.5 M NaCl solution, and precipitated overnight (12 h) at 4°C. The mixture was centrifuged at 5,000 x *g* for 15 min, and the pellet resuspended in 1 ml TBS buffer (50 mM Tris–HCl, pH 7.4, supplemented with 150 mM NaCl). After additional centrifugation at 13,000 x *g* for 5 min, the supernatant was transferred to a new centrifuge tube and reprecipated with 1/6 volume of PEG/NaCl (20% (w/v) PEG 6000+ 2.5 M NaCl) for 1 h at 4°C. The precipitated phages were recovered by centrifugation at 13,000 x g for 8 min. The pellet was resuspended in 1 ml TBS buffer (50 mM Tris–HCl, pH 7.4; supplemented with 150 mM NaCl) and centrifuged again at 13,000 × *g* for 8 min. The supernatant was stored at 4°C before further use.

Two different methods were used to detect active bacteriophages. A culture was concentrated to a final OD_600_ of 1.1 in a total volume of 10 ml fresh medium containing 20 mM methanol. One ml of the precipitated phage suspension described above or 1 ml supernatant from a centrifuged outgrown culture (8,000 x *g*; 10 min) was added to the culture, and further growth was followed measuring OD_600_.

Primers were designed to amplify a specific major capsid protein (Tph_c23140) which was found in the genome (gep3034F: ACGCGGGAACGACGGACTG and gep3034R: CGGCGGGCGAACTCTTTG). The PCR reaction was performed as follows: 1× PCR buffer, 1.5 mM MgCl_2_, 0.1 mM dNTPs, 1 U *Taq* polymerase (all Invitrogen), 5 μM of each primer, and sterile UV-irradiated water to give a final volume of 25 μl. One μl of extracted DNA of *Clostridium pasteurianum* or 1 μl phage solution was added to two separate aliquots of this mixture and the reactions were heated to 94°C for 3 min, followed by 30 cycles of 94°C for 60 s, 55°C for 50 s and 72°C for 90 s. A final extension was carried out at 72°C for 10 min. The 16S rDNA gene was amplified to control whether the phage solutions were contaminated with genomic DNA. The following primers were used for 16S rDNA gene amplification 27f: GAGTTTGATCMTGGCTCAG and 1492R: GGTTACCTTGTTACGACTT. The PCR reaction was carried out as described above.

### Analysis of cytochromes

Cell-free extract was prepared under oxic condition from a pure culture grown on methanol plus CO, or formate. Redox difference spectra (dithionite-reduced minus air-oxidized) were recorded with a spectrophotometer (Uvikon 930, Kontron Instruments).

### Chemical analyses

All substrates and products were analyzed by HPLC. Sulfate and thiosulfate were separated on an anion separation column (LCA A03, Sykam) with an eluent containing 5 mM NaHCO_3,_ 400 μM 4-hydroxybenzonitril, and 10% (v/v) acetonitril at 40°C. Conductivity was measured with the S3115 conductivity detector (Sykam). Formate, methanol, and acetate were separated on an anion separation column (Aminex HPX-87H, Biorad) with a 5 mM sulfuric acid eluent at 40°C, and measured with a refraction detector (RID-10A, Shimadzu).

### Electron microscopy

Cells were harvested by filtration through a 0.4 μm PTFE Filter. The retentate was resuspended in 200 μl 50 mM Tris–HCl buffer, pH 8.0, and fixed with 2.5% glutardialdehyde in 0.1 M Na-Cacodylate. Ten mM CaCl_2_ and 10 mM MgCl_2_ were added. Samples were filtered on a 1 μm PC membrane and washed with 30, 50, 70 and 90% ethanol. Afterwards, samples were dried by critical-point dehydration with carbon dioxide, followed by sputtering (Baltec SCD 10) with 5 nm gold-palladium. The Auriga cross-beam work station (Zeiss) was used for analysis.

## Results and discussion

### General genome features

The completed genome of *Th. phaeum* (accession number CP003732) comprises a single circular chromosome of 2,939,057 bp and an overall GC content of 53.88 mol%. Three complete rRNA clusters and 51 tRNA genes, including those for selenocystein incorporation, were identified in the genome. Approximately 75% of the open reading frames (ORFs) could be functionally annotated. The remaining 645 ORFs are made up of hypothetical proteins (631) or pseudogenes (14). 2165 (app. 73%) of all predicted protein-encoding genes could be allocated to the 21 functional COGs (Cluster of Orthologous Groups). This is in the same range as described for other acetogenic bacteria such as *Acetobacterium woodii* WB1 and *Moorella thermoacetica* ATCC39073, or sulfate-reducing bacteria such as *Desulfobacterium autotrophicum* HRM2, *Thermotoga lettingae* TMO, and *Desulfotomaculum kuznetsovii*. Analysis of COG revealed that ~34% of all protein-encoding genes fall into four main categories: amino acid transport and metabolism (10%), replication, recombination and repair (9%), energy metabolism (8%), and coenzyme transport and metabolism (7%).

**Figure 1:**
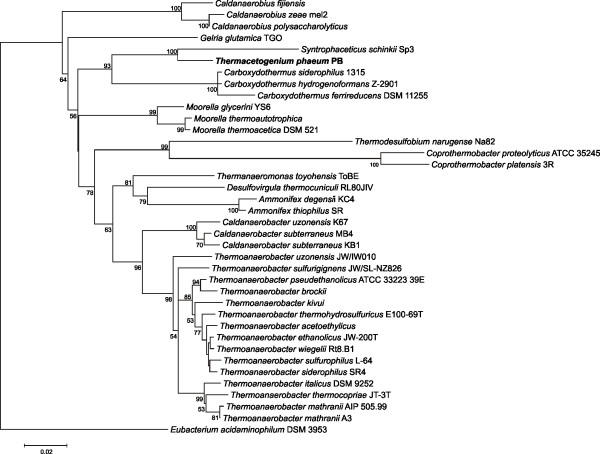
**Maximum likelihood tree of selected *Thermoanaerobacterales *type strains:** Phylogenetic calculation based on 16S rRNA genes was performed with MEGA5 (Molecular Evolutionary Genetics Analysis). The alignment was calculated with ClustalW [46]. Based on this alignment a maximum likelihood tree was inferred with 100 bootstrap replicates. 16S rRNA genes of selected *Thermoanaerobacterales* type strains were used for analysis.

*Th. phaeum* is a member of the order *Thermoanaerobacterales* within the very large and diverse phylum of the *Firmicutes*. A 16S rRNA-based phylogenetic analysis (Figure 1) of all type strains of the order *Thermoanaerobacterales* revealed that *Syntrophaceticus schinkii* is the closest known relative of *Th. phaeum*. Like *S. schinkii, Th. phaeum* is known as a syntrophically acetate-oxidizing bacterium able to oxidize acetate in coculture with a hydrogenotrophic methanogen. In contrast to the mesophilic *S. schinkii* which shows no significant growth above 40°C, *Th. phaeum* is thermophilic with a growth optimum at 58°C. Both bacteria also differ clearly in their substrate utilization patterns [13,14].

Up to date, only 36 different genomes of this order are publicly available (NCBI Genbank and IMG databases). The overall G+C content within this group varies between 34 and 59 mol%. With 54 mol%, *Th. phaeum* exhibits the third highest G+C content; only *M. thermoacetica* ATCC 39073 and *Ammonifex degensii* KC4 have higher G+C contents of 56 and 59 mol%, respectively. Like *Th. phaeum,* both these bacteria were described to be thermophilic, with growth optima above 50°C. The genomes of all *Thermoanaerobacterales* vary between 1.4 Mb and 3.3 Mb. With *Coprothermobacter proteolyticus* DSM 5265 being the smallest and *Thermanaeromonas toyohensis* ToBE DSM 14490 being the largest member of the class, *T. phaeum* matches very well with a size of 2.9 Mb. Phylogenetic analysis based on 16S rRNA gene sequences revealed that the closest relatives of *T. phaeum* are members of the genera *Caldanaerobius*, *Carboxydothermus*, and *Moorella*. Comparison of all inferred proteins of *T. phaeum* with the proteins of all sequenced *Thermoanaerobacterales* is in good agreement with the phylogenetic relationship inferred by 16S rDNA analysis. *T. phaeum* shows the highest number of orthologues (1516) to *M. thermoacetica*, another homoacetogenic organism.

### CRISPR defense system

CRISPRs (**C**lustered **R**egularly **I**nterspaced **S**hort **P**alindromic **R**epeats) are widespread in many bacterial and almost all archaeal genomes [49]. The CRISPR/*cas* system is a prokaryotic defense mechanism and provides immunity against invading mobile genetic elements such as phages and plasmids in an RNA interference-like manner. CRISPR loci typically consist of different numbers of non-contiguous repeats with lengths ranging from 20 to 47 bp [50] and unique spacers of different length and sequence between the repeats. Spacers arise from the integration of invader sequences that are integrated into CRISPS loci and act as immunity memory of past invasive elements [51]. CRISPR-associated sequence (*cas*) genes are often directly adjacent to the CRISPR loci.

In the genome of *Th. phaeum,* we identified two operons that encode Cas proteins. The first operon (Tph-c24370-Tph_c24580) consists of genes encoding two metal-dependent nucleases (*cas1* and *cas2)* that represent the universal core Cas genes, one RecB family exonuclease protein (*cas4*) and several proteins of the RAMP (**r**epeat-**a**ssociated **m**ysterious **p**roteins) superfamily. In addition, we identified a *cas3’* gene coding for an HD-nuclease and a *cas8b* gene coding for zinc-finger domain-containing proteins within the first operon. Cas3-type proteins are characteristic for all type I CRISPR-Cas systems, and the presence of *cas8b* within this operon allowed a further classification of this operon as the I-B/Tneap-Hmari subtype, according to the polythetic classification of CRISPR-Cas systems. The second operon (Tph_c18950-Tph_18980, Tph_c19070) also consists of *cas1*, *cas2*, *cas4*, additionally a slightly distant located *cas6* and a gene coding for a Csa1 family protein. The composition of the operon allows to allocate it to the I-A/APERN subtype. However, the classification is uncertain as a type I CRISPR-Cas systems-specific *cas3* is missing, as well as *cas8a*, the signature gene that allows the classification as a I-A/APERN subtype [52].

In addition to the two mentioned *cas* operons, we detected 11 CRISPR loci in the genome of *Th. phaeum*. An analysis of 2091 completely sequenced prokaryotic genomes from the NCBI Genbank database (as of 2012-07-04) revealed that CRISPRs are present in about 87% of archaeal genomes (105 genomes), but only in 55% of bacterial genomes (980 genomes). In all CRISPR-containing organisms the number of CRISPR loci varies between one and 21. One third of organisms contain one locus and approximately 75% harbor between one and three CRISPR loci. A small group of 28 prokaryotic organisms (~4%), including *Th. phaeum*, contain ten or more CRISPR loci. To date, *Arthrospira platensis* strain NIES 39 contains the highest number of different CRISPR loci (21). An analysis of the isolation sites and habitats of the aforementioned 28 species revealed that the majority are either thermophiles (e.g. *Th. phaeum*), hyperthermophiles, acidophiles, or halophiles (except for six species). These results may indicate that especially organisms living in extreme habitats very often have to cope with mobile genetic elements and the resulting horizontal gene transfer. Extreme habitats may require horizontal gene transfer and its regulation to adapt to fast environmental changes. As described above, we could identify 11 CRISPR arrays in the genome of *Th. phaeum*. The number of direct repeats within the arrays varies between eight in the smallest loci and 105 and 113 in the two largest loci. Compared to other CRISPR-containing organisms, the extreme range of direct repeats is unusual. Our analysis discovered that only 9.3% of all organisms sequenced so far contain CRISPR arrays with more than 100 repeats.

### Substrate tests and pathway construction

The information obtained from the sequenced and annotated genome on substrate degradation was counterchecked with growth experiments with *Th. phaeum* and compared with results of previous growth experiments. In addition to prior known growth substrates, we also observed growth with carbon monoxide. It was previously shown that *Th. phaeum* can grow independent of sodium ions in sodium-free media with pyruvate as substrate [53]. This was confirmed with other growth substrates (CO, formate, methanol), down to a limit of about 100 μM Na^+^. *Th. phaeum* grew with methanol, acetoin, 2,3-butanediol, and ethanol in pure culture; and in syntrophic co-culture with acetoin and, rather weakly, with 2,3-butanediol and ethanol (Table 1). No growth was observed with syringate and trimethylamine.

**Table 1:** **Growth of *Thermacetogenium phaeum *with different substrates in pure and syntrophic co-culture**

**Pure culture**	**ΔOD**_**600**_	**Cell density [mg · l**^**-1**^**]**	**Substrate utilized [mM]**	**Acetate formed [mM]**	**electron balance [%]**	**Molar growth yield Y**_**E**_**[g · mol**^**-1**^**]**
Acetoin	0.05	12.5	7.3	10.5	97.5	1.71
2,3 Butanediol	0.046	11.5	4.5	8.3	151.3	2.56
Methanol	0.583	145.8	12.2	7	86.5	11.95
Ethanol	0.047	11.75	15.3	17.2	75.5	0.77
**Syntrophic culture**						
Acetoin	0.377	94.25	7.4	12.5	128.5	12.74
2,3 Butanediol	0.045	11.25	7.7	11.4	120.1	1.46
Methanol	0.02	5	0.2	0.2	-	-
Ethanol	0.07	17.5	20.2	15.7	52.3	0.87

Molar growth yields (Y) and electron balance were calculated using the formula <C_4_H_7_O_3_> for cell material and the experimentally determined conversion factor OD600 = 1 = 0.250 g cell mass· L^-1^**.**

Data show growth after 4 weeks. In most cases no further growth could be observed.

### Sulfur metabolism

Cysteine is the primary source of sulfur for *Th. phaeum*, and is required especially for Fe-S cluster formation. There are three different *L*-cysteine degradation pathways known which all form pyruvate as an intermediate, but none of the key enzymes (*L*-cysteine:oxygen oxidoreductase, *L*-cysteine desulfhydrase, *L*-cysteine aminotransferase) was found in the genome of *Th. phaeum*. We identified four different cysteine desulfurase genes (Tph_c09490, Tph_c17300, Tph_c17960, Tph_c19200). One of them is located in a nif-like operon which is probably involved in the formation of the Fe-S cluster of nitrogenase. The other three genes are dispersed in the genome, and the gene neighbourhood shares no similarity to other well-known operons involved in Fe-S cluster assembly, such as the *suf* or the *isc* operon [54,55].

According to its original description, *Th. phaeum* can reduce sulfate, but we could not reproduce this result. Weak growth with acetate plus sulfate was observed (an OD rise from 0.04 to 0.08) but never increased further, even after several months of incubation or substrate addition. Measurement of sulfate with BaCl_2_ solution or HPLC analysis revealed that the sulfate concentration did not decrease. The only hint towards sulfate metabolism in the genome was a sulfate permease (Tph_c27320), but key enzymes of sulfate reduction such as sulfate adenyltransferase or APS reductase were not found. Interestingly, there were two thiosulfate reductase genes (Tph_c01240, Tph_c01280), but growth with thiosulfate could not be observed, and genes necessary for sulfite reduction were not found either.

### Cofactors and vitamins

Tetrahydrofolate is the most important cofactor in the Wood-Ljungdahl pathway. A closer look at the pathway of tetrahydrofolate biosynthesis revealed that all required genes despite one were present. The dihydrofolate reductase gene is missing in the genome. BLASTP searches with known sequences of dihydrofolate reductases against the *Th. phaeum* genome revealed no proper candidate for such an enzyme. However, it was shown that a dihydropteridine reductase [EC 1.5.1.34] of *Thermus thermophilus* showed 20% activity with dihydrofolate as substrate [56]. The metabolic role of dihydrobiopterine is not well understood; some aromatic amino acid hydroxylases use tetrahydrobiopterine as reducing agent while dihydrobiopterine is regenerated by dihydropteridine reductase [57]. Thus, it is likely that the product of this gene (Tph_c13060) which shows similarities to that of dihydropteridine reductase fulfills the role of the dihydrofolate reductase in *Th. phaeum*.

Also cobalamin is a necessary cofactor in the Wood-Ljungdahl pathway, and is involved in the activation of several methylated compounds as well. The synthesis pathway could not be fully reassembled because several enzymes are missing. Starting with *L*-glutamate, all genes for the synthesis of precorrin-2 were found. Of the two pathways for cobalt insertion, i. e., the “early” and the “late” cobalt insertion pathway [58,59], two enzymes are missing in either case. The “early” cobalt insertion path lacks putative genes encoding the precorrin-2 cobalt chelatase and the cobalt-precorrin-7 (C15)-methyltransferase genes, while the “late” one has no putative precorrin-3B synthase and precorrin-6B synthase encoding genes. Since the “late” cobalt insertion pathway requires oxygen, only the “early” cobalt insertion pathway is likely to operate in *Th. phaeum*. Most genes for this pathway are located in one operon, but possible genes for missing enzymatic reactions are dispersed in the genome such as a cobalt insertion protein (Tph_c03440) which is similar to *cobN.* This gene is found in the “late” cobalt insertion pathway which forms a heterotrimeric cobalt chelatase complex CobNST [60], but there is no indication of *cobS*- or *cobT*-like genes in the genome. Other chelatases might substitute for this function by similar reactions which are not directly linked to a cobalt chelatase, e. g., Mg chelatase (Tph_c17340, Tph_c17350, Tph_c10400) or *cysG* (Tph_c15350). CysG is a bifunctional methyltransferase and ferrochelatase which is involved in siroheme synthesis. This enzyme can also be involved in cobalamin synthesis [61] and may act in cobalt insertion [62].

Possible biosynthesis of quinones would be of interest because quinones could be involved in electron transport and proton translocation. It was reported earlier that *Th. phaeum* contains menaquinone-7 [13]. All genes necessary for menaquinone biosynthesis were found except for the 1,4-dihydroxy-2-naphthoate octaprenyltransferase. Only one candidate gene coding for an ubiA prenyltransferase-like (Tph_c02040) enzyme was found which shares no sequence similarities to known 1,4-dihydroxy-2-naphthoate octaprenyltransferase genes. Thus, it is questionable whether this gene is the missing link, but the presence of a menaquinone-7 has been proven in earlier studies [13].

No genes were found that could be involved in cytochrome biosynthesis. We also looked for the presence of cytochromes via redox difference spectral analysis after growth under different conditions but could not find any absorbance bands typical of cytochromes.

### CO_2_ fixation and wood-ljungdahl pathway

Previous experiments had shown that *Th. phaeum* reduces CO_2_ to acetate through the Wood-Ljungdahl pathway [63]. We found all genes necessary for this pathway in the genome, and most of them were encoded only once. However, there were four different gene clusters for formate dehydrogenases, three for carbon monoxide dehydrogenases, and two for formyl tetrahydrofolate synthetase genes. One formate dehydrogenase gene (Tph_c26260) is located inside a putative formate hydrogen lyase operon, with four possibly membrane-associated hydrogenase subunits. The genes for the other three formate dehydrogenases were found dispersed in the genome, but one of them (Tph_c15380- Tph_c15400) has three subunits (alpha, beta and gamma) and is located close to a siroheme biosynthesis operon. Interestingly, the gamma subunit (Tph_c15380) shares similarity with a cytochrome *b*_561_ subunit, and is very likely to be membrane-associated. None of these formate dehydrogenase has a TAT motif, thus they are not periplasmic. One of the carbon monoxide dehydrogenases is the bifunctional carbon monoxide dehydrogenase/acetyl-CoA synthase (Tph_c15170, Tph_c15180), the key enzyme of the Wood-Ljungdahl pathway. It is located in the *acs* operon which contains in addition a methyltetrahydrofolate:corrinoid/iron-sulfur methyltransferase (Tph_c15130), a corrinoid/iron-sulfur protein (Tph_c15140), and the methylenetetrahydrofolate reductase (Tph_c15100). An interesting feature of the *acs* operon is the presence of two genes resembling heterodisulfide reductase genes (Tph_c15090, Tph_c15120). There are two further carbon monoxide dehydrogenase genes (Tph_c05730, Tph_c11250) which are similar to the proton-translocating carbon monoxide dehydrogenase of *Methanosarcina barkeri*.

The genomes of three other homoacetogenic bacteria have been sequenced and annotated, i. e., *A. woodii*[28], *M. thermoacetica*[27,28], and *Clostridium ljungdahlii*[30].

*A. woodii* has no cytochromes or menaquinone, but uses an Rnf complex to establish a sodium ion gradient across the membrane [64]. During growth of *A. woodii* with H_2_ plus CO_2_, an electron-bifurcating [FeFe]-hydrogenase uses 6 mol H_2_ to reduce 3 mol NADH and 3 mol ferredoxin. Ferredoxin is reoxidised by the Rnf complex to reduce another 3 mol NAD^+^ and translocate six mol of Na^+^. The NADH is channelled into the methylene-THF dehydrogenase and methylene-THF reductase, and the latter reaction is supposed to reduce ferredoxin by a bifurcation reaction which is used further to reduce CO_2_ to CO. An enzyme complex composed of a FeFe hydrogenase, an iron-sulfur protein and a molybdenum/tungsten-dependent formate dehydrogenase catalyzes the reduction of another CO_2_ to formate [28]. This type of homoacetogenic metabolism requires a bifurcating hydrogenase [65], a sodium-pumping Rnf complex and a bifurcating methylene-THF reductase/ferredoxin reductase to conserve a fraction of an ATP equivalent per reaction with the help of a sodium-pumping ATPase.

Energy conservation during lithotrophic growth of *Moorella thermoacetica* is less clear. Current evidence suggests that energy is conserved in the corrinoid-dependent transmethylation from methyl-THF to the acetyl-CoA synthase enzyme. Further energy has to be obtained in electron transport via menaquinone, FAD or cytochrome to methylenetetrahydrofolate (methylene-THF) [66] (E_0_^`^= −200 mV [67]). *M. thermoacetica* also contains a bifurcating hydrogenase [68]. In contrast to *A. woodii *[28], *M. thermoacetica*[50] is not sodium-dependent but probably fuels its ATP synthesis through proton translocation.

Also *C. ljungdahlii* is sodium ion-independent but does not contain cytochromes. In this bacterium, a proton-pumping Rnf complex appears to play a major role in energy conservation [30].

**Figure 2:**
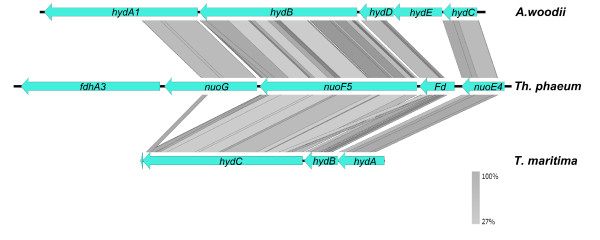
**Comparison of the putative bifurcating hydrogenase gene clusters of *Th. phaeum *with *Acetobacterium woodii *and *Thermotoga maritima.*** Linear comparison of the bifurcating hydrogenase gene loci from *A. woodii* and *T. maritima* with *Th. phaeum* was perfomed with tBlastx using easyfig [83]. The lines indicate sequence identity ranging from 27% light grey to 100% dark grey. It shows that *nuoG*, *nuoF5*, *Fd* (ferredoxin-like) und *nuoE4* are similar to the bifurcating hydrogenases of *T. maritima* and *A. woodii*. For each gene, several paralogue genes are found in the *Th. phaeum* genome; two further gene clusters are likely containing putative *hydA, hydB* and *hydC* genes (Tph_c14870-Tph_c14890 and Tph_c08240-Tph_c08260).

Based on these results, it appears that *Th. phaeum* differs from the two other metabolic types of homoacetogenic bacteria characterized above, the proton-dependent cytochrome-containing *M. thermoacetica* and the sodium-dependent *A. woodii*. In *Th. phaeum* there is no Rnf complex; only one gene (Tph_c26790) shares weak similarity to RnfC. However, *Th. phaeum* contains four genes (Tph_c18430 - Tph_c18460) which may compose a potentially bifurcating [FeFe]-hydrogenase, similar to the [FeFe]-hydrogenase of *A. woodii*[28] or *Thermotoga maritima*[69,70] (Figure 2). These genes, which have been annotated as NADH:quinone oxidoreductase, share high similarities; hence, it is tempting to predict a bifurcating hydrogenase in *Th. phaeum* which can connect to methylene-THF reduction or oxidation, either directly or indirectly via a menaquinone. Next to the putative hydrogenase, a formate dehydrogenase subunit (Tph_c18420) is located, suggesting a potential link between formate oxidation und bifurcating hydrogen formation.

In addition, four further putative hydrogenases were found in the genome. One shares similarity to a non-F_420_ reducing hydrogenase (Tph_c26910), one is similar to an Ech hydrogenase (Tph_c21310- Tph_c21360), one is connected to a formate hydrogen lyase system (Tph_c26250- Tph_c26370), and the last one is a periplasmic [NiFeSe] hydrogenase (Tph_c06350, Tph_c06360, Tph_c06370). The Ech hydrogenase and the formate hydrogen lyase are of special interest because they both could couple to proton translocation and interestingly are also found in *Moorella thermoacetica*[71,72].

Carbon monoxide dehydrogenases can use different electron acceptors such as ferredoxin or rubredoxin [73-75]. Some of these are involved in proton translocation [76]. *Th. phaeum* has three carbon monoxide dehydrogenases. One is the bifunctional carbon monoxide dehydrogenase/acetyl-CoA synthase (Tph_c15170, Tph_c15180). The other two (Tph_c05730, Tph_c11250) consist of two subunits each and are both similar to the proton-translocating carbon monoxide dehydrogenase of *M. barkeri*[76]).

Despite that, a further energetic problem has to be considered. The oxidation of methyl-THF to methylene-THF with NAD^+^ as electron acceptor is an endergonic reaction. Methylene-THF reductase (Tph_c15100) is found only once in the genome and is located in the *acs* gene cluster; a heterodisulfide reductase-homologous gene is found nearby. It was proposed that these genes and a hydrogenase could form a complex which is likely involved in proton translocation [77], but this is possible only if methylene-THF is reduced to methyl-THF. In the endergonic, oxidative direction, a proton gradient could provide the necessary energy, e. g. established through an Rnf complex.

### ATP synthase

**Figure 3:**
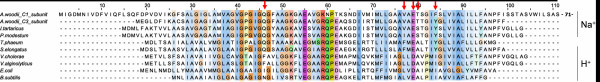
**Comparison of partial sequence alignments of the gamma subunit of F_1_F_0_-type ATPase (Tph_c27380) of *Th. phaeum *with those of other bacteria.** The following sequence accession numbers in the NCBI protein database were used: *Escherichia coli* P68699, *Vibrio cholerae* AAF95908, *Vibrio parahaemolyticus* P0A308, *Bacillus subtilis* P37815, C1 subunit *Acetobacterium woodii* (AFA47025.1), C3 subunit *Acetobacterium woodii* (AFA47026.1), *Synechococcus elongatus* (YP_399351), *Propionigenium modestum* (P21905), Ilyobacter tartaricus (Q8KRV3). Red arrow mark the position of the conserved amino acids involved in sodium binding. *T. phaeum* differs at Position 77 (phenylalanine) and 80 (alanine).

A F_1_F_0_-type ATP synthase was found in the genome which consists of 7 subunits. The gamma subunit of the F_0_-complex is involved in the proton- or sodium-pumping activity. Sequence alignments show that the gamma subunit (Tph_c27380) of *T. phaeum* shares some similarities with proton-dependent ATP synthase but also with sodium-dependent ATP synthase (Figure 3). The sodium binding motif differs slightly from other c subunit of known sodium-dependent anaerobes, but has the same motif as the proton-dependent cyanobactierum *Synechococcus elongatus*. This might explain why sodium ions at different concentrations did not impact on growth, and might indicate that the *T. phaeum* ATP synthase is proton-dependent as well.

### Flagella, gas vesicles, and bacteriophages

**Figure 4:**
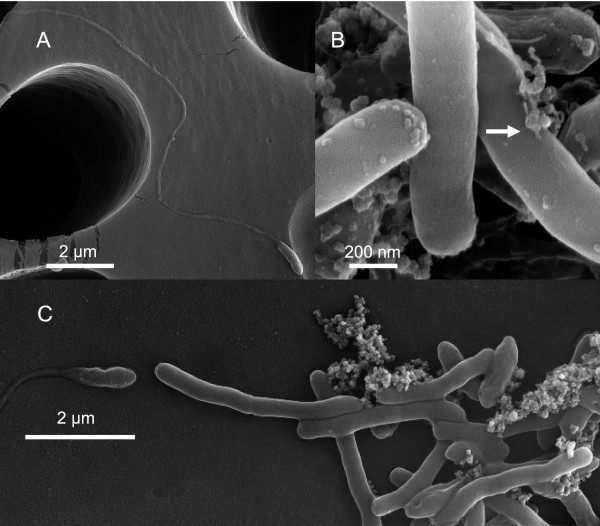
**Electron micrographs of a syntrophically grown co-culture of *Th. Phaeum andMethanothermobacter thermautotrophicus strain TM. *A** Young cell with flagellum. **B** Round structures on top of cells that might comprise bacteriophages; some show a tail-like structure (arrow)**. C** Outgrown cells forming aggregates do not carry flagella.

*Thermacetogenium phaeum* was reported to be a motile bacterium. The genome contains a complete set of flagellum genes. While the type III secretion system genes, motor/switch, basal body, and hook-associated genes are located on one operon (Tph_c10790 - Tph_c11040), the other genes involved in flagellum assembly, i. e. the filament (Tph_c22150), hook-filament junction (Tph_c05670, Tph_c05690), and filament cap gene (Tph_c22170), were found dispersed in the genome. The L and P ring genes and several transcriptional regulators were missing. Motility could explain how *Th. phaeum* and the methanogenic partner can get into close contact in order to establish an efficient syntrophic cooperation. Flagella were found by electron microscopy of young cells in syntrophic culture (Figure 4A). However, in swarm agar tubes [48] synthesis of flagella could not be induced. Additionally, a long, rod-shaped structure of 5–10 nm width and several micrometers length was found in the supernatant of syntrophic cultures which was never found in pure cultures grown with carbon monoxide.*Th. phaeum* might express a flagellum at an early stage of growth to establish close contact to a suitable partner organism. Because of their extreme energy limitation, the cells may loose the flagellum afterwards due to the high energy consumption associated with the maintenance and use of a flagellum.

The genome contains ten genes for synthesis of gas vesicles, which are located on one operon (Tph_c10500 - Tph_c10640). Four genes code for different sizes of the structural protein GvpA, which forms the main mass of the gas vesicle structure. Another structural protein GvpC was identified which enforces gas vesicle. Two types of the *gvpL/gvpF* genes and one copy of *gvpG, gvpK, gvpN* each were detected; all these genes are likely to be involved in the formation of the gas vesicle [78]. Thus, gas vesicle formation in *Th. phaeum* is genetically possible, but they were never detected in growing cultures. Additionally, floating cells could not be observed either.

**Figure 5:**
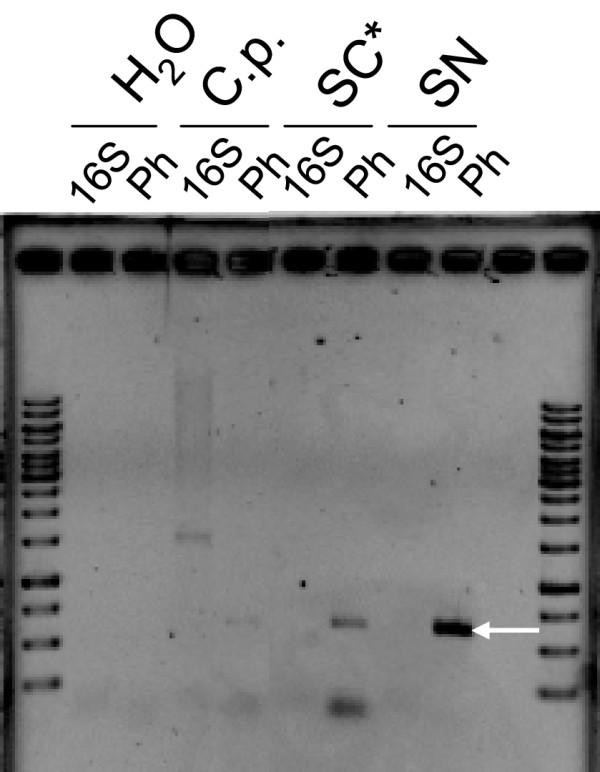
**Detection by PCR of phage-related DNA in the supernatant of a syntrophically grown culture of *Th. Phaeum *and *Methanothermobacter thermautotrophicus strain TM*.** Concentrated phage solution from a culture (SC*) and filter-sterilized culture supernatant (SN) were used. As a control for the 16S rDNA PCR, also DNA of *Clostridium pasteurianum* (C.p.) was used. A PCR product (arrow) was formed with the phage-specific primer in the supernatant (SN Ph) of the culture and in the concentrated phage solution prepared from the same supernatant (SN* Ph). A weak band is visible also in the *Clostridium pasteurianum* lane with phage-specific primer.

Another interesting feature is a set of genes (Additional file 1: Table S1) that comprise a complete bacteriophage genome. Earlier experiments had indicated that the culture may harbor a phage: During growth of five replicate cultures, always one or two cultures grew slower than the other ones although the growth conditions were exactly identical. Possible phages were concentrated by PEG/NaCl precipitation from the supernatant of such cultures, and this phage preparation was added to a dense culture at OD 1.0. While the control culture continued to grow up to OD 1.2 the culture with the phage suspension showed no growth at all. Since the phage-related genes were known, a primer pair targeting specific phage DNA was designed. PCR was employed to show that the supernatant contained phage DNA (Figure 5) which might be a result of active phages. In order to check whether the supernatant contain genomic DNA primer for 16S rDNA were used. No bands were visible which means that the band from the phage specific primer results from an active phage and not a prophage. The band in the *Clostridium pasteurianum* lane (Ph) may be an uspecific product, because no similar gene was found in the *C. pasteurianum* genome.

To confirm these results, we performed electron microscopy and found regularly shaped round structures, which might be phages (Figure 4B). Obviously, this phage impairs growth of *Th. phaeum* in pure and syntrophic culture. Unfortunately, we could not identify the trigger which activates the lytic cycle. So far, the phage appears to impair growth randomly under different growth conditions with different substrates. After severe lysis, cultures were able to recover and to resume growth again. The phage might be the reason for extremely low growth yields, especially during growth with “difficult” substrates such as CO, H_2_, or formate.

### Energy conservation during growth in pure culture

Two ways of methanol oxidation are possible. It was reported that in *Clostridium thermoautotrophicum* the oxidation of methanol via formaldehyde to formate involves a pyrroloquinoline quinone-dependent methanol dehydrogenase [79]. Other methylotrophic homoacetogens transfer the methyl group to a different methyl acceptor, which is either a corrinoid Fe-S protein or tetrahydrofolate [80,81]. *Th. phaeum* uses the second pathway, because a methanol-cobalamin methyltransferase (Tph_c03590) was found which likely activates and transfers the methyl group to a methyl acceptor. The genome contained several alcohol dehydrogenases, but a dehydrogenase activity with methanol could not be found. Fermentation of 4 mol methanol yields 3 mol acetate:

4 Methanol + 2 CO_2_ → 3 Acetate^-^ + 3 H^+^ + 2 H_2_O ΔG^0`^ = −52.3 kJ per mol methanol [82].

This fermentation yields one ATP equivalents for the oxidation of one methanol and yields three ATP in the acetate kinase reaction. Part of this ATP surplus has to be sacrificed to drive the endergonic oxidation of methyl-THF to methylene-THF (see above). To our surprise, no growth with methanol was observed in the syntrophic co-culture.

Growth with CO is energetically easy:

4 CO + 2 H_2_O **→** Acetate^-^ + H^+^ + 2 CO_2_ ΔG^0`^ = −175.0 kJ per mol.

Due to the low redox potential of the CO_2_/CO couple (E°’=−520 mV), CO oxidation can deliver electrons to all reduction steps in the Wood-Ljungdahl pathway, and net ATP formation could be fueled through proton-translocating CO dehydrogenases for which evidence was obtained in the genome. Nonetheless, growth of *Th. phaeum* with CO was often difficult to reproduce; perhaps the bacteriophage that we found in the genome is activated especially in the presence of this low-potential electron donor.

Growth experiments confirmed growth with acetoin, but growth with ethanol and 2,3-butanediol was rather poor. Methanol was used only in pure culture. No growth was observed with syringate or trimethylamine (Table 1).

According to the genomic data, 2,3-butanediol is degraded via acetoin and further cleaved to acetaldehyde plus acetyl-CoA, with NAD^+^ as electron acceptor. Acetaldehyde can be oxidized to acetate, either by an acetylating aldehyde dehydrogenase, phosphate acetyltransferase and acetate kinase, or be oxidized by a non-acetylating acetaldehyde dehydrogenase directly to acetate. In the first pathway, a total of 2 ATP and 2 NADH is formed. The only NADH-dependent hydrogenase found in the genome is the putatively bifurcating [Fe]-hydrogenase. The other pathway forms one NADH, one reduced ferredoxin, and ATP. This pathway might be more likely because the reduced ferredoxin is already available. Comparing growth of the syntrophic co-culture and the pure culture with acetoin, it appears that hydrogen formation is the energy-limiting step. Substrate utilization and product formation in both growth modes are similar, but the syntrophic culture reaches a far higher OD. Interestingly, there is no difference between the pure and the syntrophic culture during growth on 2,3-butanediol, indicating that in this case the methanogen does not help by syntrophic electron removal. The same applies to oxidation of ethanol to acetate: we found nearly quantitative substrate conversion but only very weak growth. So far, we have no explanation for this observation.

It was also reported that *Th. phaeum* grew with syringate or vanillate. Several o-methyltransferase-like proteins (Tph_c05610, Tph_c22260) and tri-/dimethylamine methyltransferases (Tph_c27660- Tph_c27720, Tph_c05860, Tph_c05880) were found in the genome which may transfer the methyl group of the corresponding substrate to tetrahydrofolate and ferment it analogous to methanol. However, no growth was observed on syringate or trimethylamine either.

### Syntrophic growth with acetate

*Th. phaeum* degrades acetate syntrophically and forms H_2_ and CO_2_ (and possibly formate) which can be used further by the methanogenic partner to form methane.

CH_3_COO^-^ + H^+^ + 2 H_2_O **→** 2 CO_2_ + 4 H_2_ ΔG^0`^ = + 95 kJ per mol rct.4 H_2_ + CO_2_**→** CH_4_ + 2 H_2_O ΔG^0`^ = −131 kJ per mol rct.CH_3_COO^-^ + H^+^ + **→** CH_4_ + CO_2_ ΔG^0`^ = −35 kJ per mol rct.

The final acceptor for the released electrons is H^+^ (E_0_^`^ = −300 mV [15], pH 7.0, 10 Pa H_2_) at a redox potential lower than the methylenetetrahydrofolate/methyltetrahydrofolate couple. *Th. phaeum* uses the Wood-Ljungdahl pathway for CO_2_ fixation and acetate oxidation. An interesting question is whether the same enzymes are involved under both growth conditions. Besides formate dehydrogenase, CO dehydrogenase, and hydrogenase, only the formyl-THF synthetase has a further paralogue gene in the genome (Tph_c08280, Tph_c26780). As discussed above, formate dehydrogenase, CO dehydrogenase, methylene-THF reductase and hydrogenase are of special interest concerning the energy conservation in both directions.

Based on our data it is likely that different enzymes are expressed under different growth conditions to allow ATP synthesis in either direction. Only methylene-THF reductase is present only once. The different pathways of energy conservation will be subject to further studies in our lab.

## Competing interests

The author declares there are no competing interests.

## Authors' contribution

DO, AP, AL and NM did most of the gene annotations, RD and GG contributed further to gene annotations, AP did the genome sequencing, DO provided the experimental data, A.P. and D.O. prepared the figures, and DO, AP and BS wrote the manuscript. All authors read and approved the final manuscript.

## Supplementary Material

Additional file 1 **Table S1.** List of putative prophage genes. Click here for file
