# Supplementary material for: Genome-guided analysis of physiological and morphological traits of the fermentative acetate oxidizer Thermacetogenium phaeum
Source: BMC Genomics. 2012 Dec 23;13:723. doi: 10.1186/1471-2164-13-723 (PMC3551663; doi:10.1186/1471-2164-13-723)
Supplement: Additional file 1 — Table S1. List of putative prophage genes. [file 1471-2164-13-723-S1.doc]

**Table S1: List of putative prophage genes.**

| **Locus Tag** | **Annotation** |
| --- | --- |
| Tph_c23040 | phage minor structural protein |
| Tph_c23050 | phage tail component, N-terminal |
| Tph_c23060 | putative minor tail protein Gp26 |
| Tph_c23070 | hypothetical protein |
| Tph_c23080 | hypothetical protein |
| Tph_c23090 | phage major tail protein, phi13 family |
| Tph_c23100 | hypothetical protein |
| Tph_c23110 | phage protein, HK97 gp10 family |
| Tph_c23120 | putative phage head-tail joining protein |
| Tph_c23130 | putative DNA packaging protein, QLRG family |
| Tph_c23140 | phage major capsid protein, HK97 family |
| Tph_c23150 | clpP: peptidase S14 ClpP (EC 3.4.21.92) |
| Tph_c23160 | phage portal protein, HK97 family |
| Tph_c23170 | hypothetical protein |
| Tph_c23180 | phage terminase |
| Tph_c23190 | phage integrase (EC 2.7.7.-) |
| Tph_c07400 | phage integrase (EC 2.7.7.-) |
| Tph_c07410 | phage integrase (EC 2.7.7.-) |
